# Adherence to Lifestyle Medicine and Sleep-Related Problems Among First-Year University Students in Quito, Ecuador, 2024–2025: A Cross-Sectional Study

**DOI:** 10.3390/healthcare14182891

**Published:** 2026-09-08

**Authors:** Doménica Yukling León-Chang, Camila Miño, Rodrigo Yáñez-Sepúlveda, José Adrián Montenegro-Espinosa, Fiorella Quiroz-Cárdenas, Jorge Olivares-Arancibia, Emily Cisneros-Vásquez, José Francisco Tornero-Aguilera, Dong Keon Yon, José Francisco López-Gil

**Affiliations:** 1 School of Medicine, Universidad Espíritu Santo, Samborondón 0901952, Ecuador; domeleon@uees.edu.ec; 2 Faculty of Health Sciences, Universidad Tecnológica Atlántico Mediterráneo—UTAMED, 29590 Málaga, Spain; 3 European Institute of Higher Studies, IEES, 4820-509 Fafe, Portugal; camilamino501@gmail.com; 4 Faculty of Education and Humanities, School of Sport Sciences, Universidad Andres Bello, Viña del Mar 2520000, Chile; rodrigo.yanez.s@unab.cl; 5 Universidad Autónoma del Paraguay, Asunción 001010, Paraguay; josemontenegro733@gmail.com; 6 Vicerrectoría de Investigación y Postgrado, Universidad de Los Lagos, Osorno 5290000, Chile; fiorella.quiroz@yahoo.com; 7 Department of Health Research, ICEN Cognis, 38370 La Matanza de Acentejo, Spain; 8 AFySE Group, Research in Physical Activity and School Health, School of Physical Education, Faculty of Education, Universidad de las Américas, Santiago 8370150, Chile; 9 Universidad Internacional para el Desarrollo (UNINDE), 06005 Badajoz, Spain; emily.sofy@hotmail.com; 10 Department of Sport Sciences, Faculty of Sport and Health Sciences, Fit Generation Research Institute, AD500 Andorra la Vella, Andorra; jtornero@fitgeneration.es; 11 Center for Digital Health, Medical Science Research Institute, College of Medicine, Kyung Hee University, Seoul 02447, Republic of Korea; yonkkang@gmail.com; 12 Department of Pediatrics, Kyung Hee University Medical Center, College of Medicine, Kyung Hee University, Seoul 02447, Republic of Korea; 13 Faculty of Health Sciences, Universidad Autónoma de Chile, Temuco 4810101, Chile

**Keywords:** lifestyle factors, sleep quality, young adults, cross-sectional study, U-SMILE, Ecuador, DSM-5 Level 1

## Abstract

Background/Objectives: Sleep-related problems are common among university students and are linked to adverse academic and health outcomes. This study evaluated the association between lifestyle medicine adherence and sleep-related problems among first-year Ecuadorian university students. Methods: A cross-sectional study was conducted in Quito, Ecuador, during the 2024–2025 academic year. First-year undergraduate students aged 16–35 years were recruited through a census-based, non-probability strategy (*N* = 2007). Lifestyle medicine adherence was assessed using the Short Multidimensional Inventory Lifestyle Evaluation for University Students (U-SMILE), and sleep-related problems were defined using the Diagnostic and Statistical Manual of Mental Disorders, Fifth Edition (DSM-5) Level 1 Cross-Cutting Symptom Measure sleep domain (score ≥ 2), a symptom screen that is conceptually related to, but not identical with, the U-SMILE sleep quality domain. Multivariable binary logistic regression models were used and adjusted for sociodemographic and health-related covariates. A post-hoc sensitivity analysis recomputed the global U-SMILE score after excluding the sleep quality domain. Results: The median age was 18.0 years, and 51.0% were female. Among participants with Level 1 sleep data (*n* = 1906), 47.8% were classified as having sleep-related problems. In the fully adjusted model (*n* = 1893), higher overall U-SMILE scores were associated with lower odds of sleep-related problems (odds ratio (OR) per 1 point = 0.93, 95% Confidence Interval (CI): 0.91–0.94; OR per 1 SD = 0.56, 95% CI: 0.50–0.62). In a post-hoc sensitivity analysis, this association persisted after excluding the sleep quality domain from the global score (OR per 1 point = 0.94, 95% CI: 0.93–0.95; OR per 1 SD = 0.66, 95% CI: 0.60–0.73). Among non-sleep domains, the strongest associations were avoidance of substance use (OR = 0.87), social relationships (OR = 0.90), and nutrition (OR = 0.90); physical activity, nature engagement, and stress management were also significant. Conclusions: Higher lifestyle medicine adherence was associated with lower odds of sleep-related problems, including in post-hoc analyses when the sleep domain was omitted from the U-SMILE total. These findings support multidimensional lifestyle medicine approaches for sleep health promotion in university settings.

## 1. Introduction

Sleep-related problems are a frequent health condition among university students, and they represent a concern for both academic performance and overall health [1]. Chronic sleep deficiency has been associated with the onset or worsening of conditions, including obesity, diabetes, cardiovascular disease, cognitive decline, and depression [2]. A consensus statement from the American Academy of Sleep Medicine (AASM) and Sleep Research Society recommends 7–9 h of sleep per night for adults to maintain optimal health [3]. However, available data suggest that university students do not meet this recommendation [4,5,6,7]. A meta-analysis by Gardani et al. [4] showed that approximately 30% of university students meet the criteria for insomnia symptoms. According to the American College Health Association-National College Health Assessment (ACHA-NCHA) spring 2025 report, only 42% of students reported sleeping more than 7 h per night [5]. In Ecuador, evidence from a recent study of 1118 university students in Guayaquil showed that 71.2% were classified as having poor sleep quality based on the Pittsburgh Sleep Quality Index (PSQI) [6].

Lifestyle Medicine (LM) is an evidence-based approach focused on lifestyle behaviors for the prevention and management of chronic diseases [8]. The American College of Lifestyle Medicine (ACLM) identifies six core pillars: optimal nutrition, physical activity, stress management, restorative sleep, social connectedness, and risky substance avoidance [9]. However, a multidimensional approach has incorporated an additional domain, such as environmental or nature engagement [10]. These pillars are considered modifiable lifestyle behaviors that might influence overall well-being [9,10]. Instead of targeting isolated risk factors, LM emphasizes the interaction between behavioral, psychosocial and environmental factors [8,9]. In a narrative review, Rippe [11] highlighted the potential role of LM interventions in the prevention and management of chronic diseases, including coronary heart disease, diabetes, obesity, and cancer.

Previous studies have associated LM with sleep-related problems. Multidimensional LM interventions have been associated with improved sleep quality [12], while unhealthy lifestyle behaviors, such as alcohol consumption, have been linked to sleep-related problems [13]. Trinh et al. [13] reported a 40.1% prevalence of sleep-related problems among university students and identified associations with alcohol consumption. Other studies have associated individual LM pillars with sleep-related problems [14,15,16,17,18,19]. At the behavioral level, a systematic review found that unhealthy dietary patterns have been associated with shorter sleep duration and poorer sleep quality [14], whereas physical activity may support sleep by reducing anxiety and stress due to the release of endorphins [15]. Social and substance-related factors may also contribute, as social isolation has been linked to sleep-related problems and cognitive decline, while smoking and alcohol consumption have been associated with insufficient sleep and alterations in sleep architecture, including rapid eye movement (REM) sleep [16,17,18]. Environmental factors may be relevant as well, since greater access to green spaces has been associated with lower odds of sleep-related problems [19].

Despite existing evidence associating LM with sleep outcomes [9,11], most studies focus on individual lifestyle factors rather than an integrated framework. Comparisons across studies are further complicated by differences in populations, sleep assessment instruments, and outcome definitions. Studies may use broad symptom-screening measures or multidimensional instruments such as the PSQI, and different threshold values may be applied even when the same instrument is used. These variations affect the proportion of participants classified as having poor sleep quality and limit direct comparisons of prevalence estimates and associations across studies [4,7,13,20]. First-year students represent a particularly relevant population because the transition to university may involve greater autonomy, changes in academic demands and daily routines, the development of new peer networks, and, for some students, changes in residence and family support. These simultaneous adjustments may disrupt both sleep patterns and other lifestyle behaviors [13,20,21]. Nevertheless, evidence from Latin America is still limited among university students, and no studies have examined LM with sleep-related problems in Ecuador. Accordingly, this study adopted a multidimensional LM framework in which the seven domains of the Short Multidimensional Inventory Lifestyle Evaluation for University Students (U-SMILE) were examined in relation to sleep-related problems. The aim of this study was to evaluate the association between LM and sleep-related problems in first-year Ecuadorian university students. Understanding this relationship may help identify modifiable risk factors that could support health promotion strategies in university populations.

## 2. Materials and Methods

### 2.1. Study Design and Participants

The reporting of this cross-sectional study was guided by the Strengthening the Reporting of Observational Studies in Epidemiology (STROBE) recommendations [22]. This cross-sectional study was conducted at Universidad de las Américas (UDLA), a private university in Quito, Ecuador, during the 2024–2025 academic year. The study protocol was approved by the Comité de Ética para la Investigación con Seres Humanos de la Universidad de las Américas (CEISH-UDLA; protocol code 2024-OBS-016; approval date: 5 July 2024). The study population consisted of first-year undergraduate students between the ages of 16 and 35 years, who provided informed consent. The broad age range was used to include both traditional and non-traditional students entering their first year of university. The present study represents a cross-sectional analysis of data collected as part of the UNIversity students’ LIFEstyle behaviours and Mental health cohort (UNILIFE-M) [23]. First-year students from all academic fields were eligible to participate. Participants were excluded if they were unable to provide informed consent or complete the questionnaire due to cognitive, language-related, or other conditions that could compromise the reliability of their responses. These exclusion criteria were applied during recruitment by the trained psychology team and were based on the inability to provide informed consent or to complete the electronic questionnaire; no additional clinical or psychometric screening for cognitive or language disorders was performed. In the present study, no participants were excluded for these reasons.

A census-based, non-probability recruitment strategy was followed, in which all eligible individuals from the target population were invited to participate voluntarily. Because the study aimed to reach the full accessible population, an a priori sample size calculation was not conducted. Nevertheless, the final analytic sample provided adequate statistical precision for multivariable logistic regression: among participants with complete data for the fully adjusted models (*n* = 1893), approximately 48% met the sleep-related problems criterion, corresponding to roughly 909 outcome events and an events-per-variable ratio well above conventional thresholds for stable logistic regression estimation. Invitations were extended to students from different faculties and degree programs to capture a broad range of academic backgrounds and student profiles. A total of 3602 students enrolled in first-year programs and were invited to participate. Ultimately, only 2007 students responded to the questionnaire, yielding a response rate of 55.7%. A formal comparison between respondents and nonrespondents could not be performed because data on nonrespondents were unavailable. Recruitment activities were coordinated by a team of psychologists trained in the study protocol, who followed standardized procedures across faculties. The questionnaire was administered electronically to participants using Research Electronic Data Capture (REDCap; Vanderbilt University, Nashville, TN, USA). This strategy was implemented to increase response rates while maintaining consistency in data collection. Written informed consent was obtained from all participants before answering the questionnaire.

### 2.2. Variables

#### 2.2.1. Lifestyle Medicine

The Short Multidimensional Inventory Lifestyle Evaluation for University Students, U-SMILE [10], a condensed version of the Short Multidimensional Inventory on Lifestyle Evaluation (SMILE), validated in university populations, was employed to assess dimensions corresponding to the pillars of LM. A total of 24 items across seven domains were assessed in this study, including: (1) nutrition, (2) avoidance of substance use, (3) physical activity, (4) stress management, (5) sleep quality, (6) social relationships, and (7) engagement with the natural environment. The U-SMILE has demonstrated acceptable internal consistency in the present sample (Cronbach’s α = 0.74; McDonald’s ω = 0.75; *n* = 2000 participants with complete item responses), comparable to the original validation among university students (Cronbach’s α = 0.73; McDonald’s ω = 0.79) [10]. Item responses are summed across 24 items to a global score ranging from 0 to 96; higher global and domain scores indicate healthier lifestyle patterns.

#### 2.2.2. Sleep-Related Problems

Sleep-related problems were assessed using the Diagnostic and Statistical Manual of Mental Disorders, Fifth Edition (DSM-5) Level 1 Cross-Cutting Symptom Measure for Adults [24], a self-reported screening tool that assesses 13 psychiatric symptom domains: depression, mania, anxiety, sleep problems, anger, somatic symptoms, psychosis, memory problems, suicidal ideation, repetitive behaviors and thoughts, personality functioning, substance use, and dissociation, and that has been evaluated in university students. The Level 1 sleep item asks how much the respondent has been bothered, over the past two weeks, by problems with sleep that affected sleep quality overall (for example, problems falling asleep, staying asleep, or waking too early). A cut-off score of ≥2 in the sleep domain was considered positive for sleep-related problems and was used as the primary dichotomous outcome in all analyses [23,24]. This symptom screen is distinct from the U-SMILE sleep quality domain, which indexes habitual sleep duration, regularity, and sleep quality behaviors rather than current sleep-problem bother.

#### 2.2.3. Covariates

Sociodemographic characteristics considered in the analysis were: sex (male or female), age (in years, continuous), sexual orientation (non-heterosexual or heterosexual), ethnicity/race (non-Mestizo or Mestizo), marital status (non-single or single), work status (yes = employed, or no = unemployed), student accommodation (yes = living inside university, or no = living outside university), and field of study (non-health sciences or health sciences).

Among the health-related covariates, body mass index (BMI) was estimated using self-reported height and weight and then grouped according to standard categories [25]: underweight (<18.5 kg/m^2^), normal weight (18.5–24.9 kg/m^2^), overweight (25.0–29.9 kg/m^2^), and obesity (≥30.0 kg/m^2^) [25], and the presence of any mental disorder (yes/no) and any physical health condition (yes/no), both based on self-reported diagnoses.

### 2.3. Statistical Analyses

All statistical analyses were conducted in Python 3.13.7 (Python Software Foundation, Wilmington, DE, USA). Logistic regression models were fitted using ‘statsmodels’ (version 0.14.6), and data management/statistical computations were performed with ‘pandas’ (version 2.3.3), ‘NumPy’ (version 2.3.5), and ‘SciPy’ (version 1.17.0). Statistical significance was set at *p* < 0.05.

Data normality of continuous variables was assessed using statistical (Shapiro–Wilk test) and visual procedures (density plots and quantile-quantile [Q-Q] plots). Descriptive statistics were used to summarize population characteristics. Categorical variables were expressed as frequencies (*n*) and percentages (%), while continuous variables as median and interquartile range (IQR) (due to skewed distributions).

Binary logistic regression analyses, including unadjusted and multivariable adjusted models, were performed with sleep-related problems defined as a DSM-5 Level 1 sleep-domain score ≥ 2. The primary analyses evaluated the association between overall adherence to LM (i.e., global U-SMILE score) or each of the six non-sleep LM domains and sleep-related problems. Three different models were performed in each case: model 0 (unadjusted), model 1 (adjusted for age and sex), and model 2 (adjusted for age, sex, BMI status, sexual orientation, ethnicity, marital status, work status, student accommodation status, field of study, mental disorder status, and physical condition status). Age was included as a continuous covariate in Models 1 and 2 to account for potential age-related differences across the 16–35-year eligibility range. Covariates included in the multivariable models were selected a priori based on theoretical relevance and prior evidence on sleep and lifestyle behaviors in university students, rather than through data-driven bivariate screening. Model estimates were reported as odds ratios (OR) with 95% confidence intervals (CI). Estimates are reported per 1-point increase and per 1-SD increase in each U-SMILE metric; the SD was the sample standard deviation of the predictor in the corresponding complete-case model sample. We additionally presented predicted-probability curves from Models 0–2 for the overall U-SMILE score and the six non-sleep domains using empirical marginal standardization (average predicted probabilities over the observed covariate distribution), with 95% CIs.

Post-hoc sensitivity analyses (not pre-specified in the original analytic plan) were conducted to address conceptual overlap between the U-SMILE sleep quality domain and the Level 1 sleep outcome. The sleep quality domain score correlated moderately with the Level 1 outcome (point-biserial correlation coefficient [r_pb_] = −0.36, *n* = 1906). We therefore recomputed the global U-SMILE score after subtracting the sleep quality domain score and repeated Models 0–2 (Table S1).

Finally, to evaluate the functional form of associations, we compared linear, quadratic, cubic, and restricted cubic spline (with 3, 4, and 5 knots) specifications for each U-SMILE metric using the Bayesian information criterion (BIC), the Akaike information criterion (AIC), and likelihood ratio tests comparing each specification with the linear model. P-for-nonlinearity was derived from these nested comparisons, and linear forms were retained when nonlinearity was not statistically supported. Analyses were performed using a complete-case approach. Logistic regression models included participants with complete data for U-SMILE, the Level 1 sleep outcome, and covariates included in each model. Therefore, fully adjusted models were restricted to individuals with complete covariate and outcome data (*n* = 1893). The same complete-case sample was used for the post-hoc sensitivity analysis excluding the sleep quality domain. No adjustment for multiple comparisons was applied for the primary domain-specific analyses, which followed the U-SMILE framework; post-hoc sensitivity findings were interpreted cautiously. However, given the exploratory nature of this cross-sectional study, findings should be interpreted cautiously due to the potential risk of Type I error.

## 3. Results

A total of 2007 first-year university students were included (median age: 18.0 years, IQR: 18.0–19.0). The sample was evenly distributed by sex, with 51.0% females. The median U-SMILE score was 65.0 (IQR: 60.0–70.0). Regarding sleep-related problems, 101 students (5.0%) had missing DSM-5 Level 1 sleep data. Among participants with classified Level 1 sleep status, 47.8% (912/1906) presented sleep-related problems (score ≥ 2), while the remainder screened negative. Table 1 presents the sociodemographic and clinical characteristics of the study population.

Table 2 shows the associations of overall LM adherence (U-SMILE score) and its six non-sleep domains with sleep-related problems across three logistic regression models. In the unadjusted analyses (Model 0), higher overall U-SMILE scores were associated with lower odds of sleep-related problems (OR = 0.92, 95% CI: 0.91–0.94, *p* < 0.001). In the fully adjusted model (Model 2; *n* = 1893), higher overall U-SMILE scores remained associated with lower odds of sleep-related problems (OR per 1 point = 0.93, 95% CI: 0.91–0.94, *p* < 0.001; OR per 1 SD = 0.56, 95% CI: 0.50–0.62). After excluding the sleep quality domain from the global score, the association was attenuated but remained statistically significant (OR per 1 point = 0.94, 95% CI: 0.93–0.95, *p* < 0.001; OR per 1 SD = 0.66, 95% CI: 0.60–0.73; Table S1). Among non-sleep domains in Model 2, lower odds were observed for avoidance of substance use (OR = 0.87, 95% CI: 0.83–0.92, *p* < 0.001), nutrition (OR = 0.90, 95% CI: 0.85–0.96, *p* = 0.001), social relationships (OR = 0.90, 95% CI: 0.87–0.93, *p* < 0.001), nature engagement (OR = 0.91, 95% CI: 0.87–0.95, *p* < 0.001), physical activity (OR = 0.93, 95% CI: 0.89–0.97, *p* = 0.002), and stress management (OR = 0.93, 95% CI: 0.88–0.99, *p* = 0.021). The complete results for Models 0–2 are presented in Table 2, and the post-hoc sensitivity analysis excluding the sleep domain is presented in Table S1.

**Figure 1:**
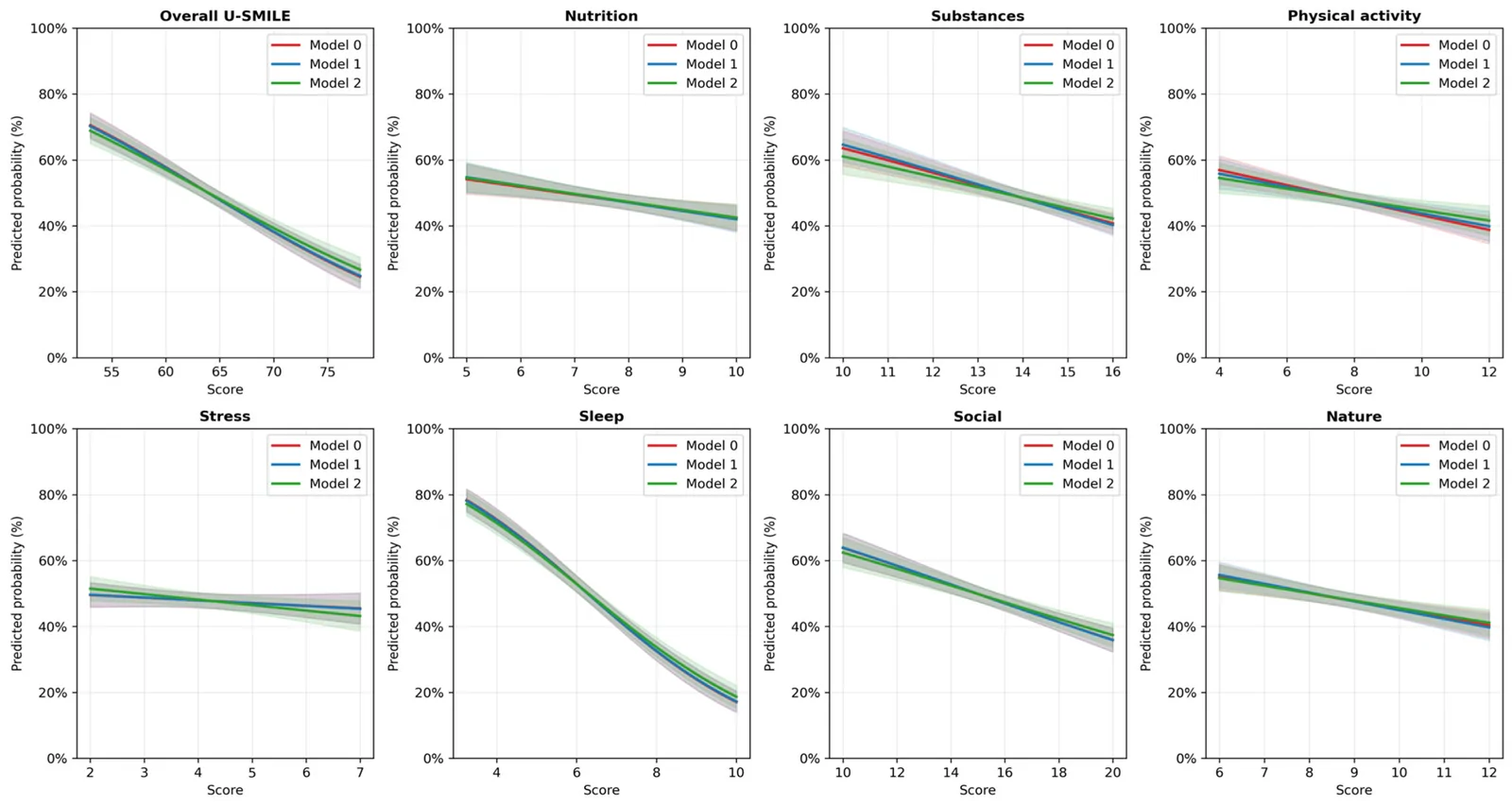
Predicted probabilities of having sleep-related problems (DSM-5 Level 1 sleep domain ≥ 2) according to the overall U-SMILE score and the six non-sleep domain scores among Ecuadorian first-year university students. Solid lines represent predicted probabilities, and shaded bands represent 95% CIs.

Figure 1 shows the predicted probabilities of having sleep-related problems according to the adherence to LM in Ecuadorian first-year university students. In the fully adjusted model (Model 2), each 1-point increase in overall U-SMILE was associated with a 7% lower odds of sleep-related problems (95% CI: 6.0% to 8.6%). At the non-sleep domain level, each 1-point increase was associated with lower odds by 9.9% (95% CI: 4.1% to 15.3%) for nutrition, 12.6% (95% CI: 7.9% to 17.2%) for substances, 6.7% (95% CI: 2.6% to 10.6%) for physical activity, 10.3% (95% CI: 7.5% to 13.0%) for social, 9.2% (95% CI: 4.8% to 13.5%) for nature, and 6.9% (95% CI: 1.1% to 12.4%) for stress management. Figure S1 presents the assessment of the functional form of the associations. No evidence of nonlinearity was observed across the models, supporting the use of linear specifications.

## 4. Discussion

### 4.1. Principal Findings

Overall, our findings indicate that higher overall U-SMILE scores were associated with lower odds of sleep-related problems, even after adjustment for sociodemographic and health-related covariates. Among the six non-sleep domains, avoidance of substance use, social relationships, and nutrition showed the strongest associations, whereas physical activity, engagement with the natural environment, and stress management showed more modest associations. These results are in line with previous evidence suggesting that sleep is related to multiple behavioral, psychosocial, social, and environmental components of LM [12]. Beyond its relationship with lifestyle behaviors, sleep has also been recognized as an important factor in cardiovascular health and mortality [26], although cardiovascular outcomes were not assessed in the present study. These findings should be interpreted within the context of the first university year, a period often characterized by changes in academic demands, daily routines, personal autonomy, and social networks, which may simultaneously influence sleep and lifestyle behaviors [20].

The main inference rests on the six non-sleep pillars. Post-hoc sensitivity analyses indicated that the global association persisted after the sleep quality domain was removed from the total score (Table S1). Previous research in university populations has shown that late-night internet use and irregular sleep schedules are associated with sleep-related problems and daytime dysfunction [13]. Sleep-hygiene behaviors are also recognized as relevant modifiable factors for sleep health [27]. Another important finding was the association between avoidance of substance use and sleep-related problems. This association may be explained by the effects of substances such as alcohol, caffeine, and nicotine on circadian regulation and sleep architecture [17,18]. For example, although alcohol may initially promote sleep onset, it subsequently impairs REM sleep and increases nocturnal awakenings [18]. A similar association between alcohol consumption and sleep-related problems has been reported among first-year university students [13].

Similarly, social relationships were linked with lower odds of sleep-related problems. Previous evidence also supports an association between social relationships and sleep outcomes [28,29,30,31]. A cross-sectional study by Dong et al. [28] reported that a higher level of loneliness is associated with decreased sleep quality in university students, mediated by perceived stress and expressive suppression. The potential protective role of social relationships could be explained through some psychobiological pathways. For instance, social support reduces the activation of the hypothalamic–pituitary–adrenal axis, which leads to improved stress regulation and, consequently, lower cortisol levels [31]. Additionally, individuals with weaker social ties may be more likely to engage in unhealthy behavior [30], such as substance use and irregular lifestyle patterns, which may further contribute to sleep-related problems. Beyond the social environment, the natural environment may also be relevant to sleep through related stress-regulation pathways. A meta-analysis by Shin et al. [19] suggests that adults with higher exposure to green areas have lower odds of experiencing sleep-related problems. A possible mechanism underlying this relationship involves stress reduction, as from a psychophysiological perspective, Twohig-Bennett et al. [29] linked nature exposure to reduced stress levels and lower cortisol secretion, which may facilitate better sleep quality.

Another relevant finding of this study was that higher physical activity and nutrition scores were associated with lower odds of sleep-related problems. Previous studies have reported associations between physical activity and better sleep quality; however, these associations may vary according to exercise timing, intensity, and individual characteristics [32,33]. A meta-analysis by Stutz et al. [33] concluded that exercise improves sleep efficiency and duration, but its effect depends on the factors previously mentioned. Supporting this notion, physical activity may be related to sleep through endorphin-mediated pathways involved in stress and anxiety regulation [15]; however, late high-intensity exercise can increase stimulation by sympathetic activation and elevated cortisol levels, which can delay sleep onset and impair sleep quality [33]. In addition, Godos et al. [14] found that higher consumption of plant-based foods, healthy fats like omega-3, and the type of protein (i.e., plant-based protein vs. animal protein) were related to lower odds of sleep-related problems. This could be explained by the role of dietary patterns in modulating inflammation pathways [34], which has been related to poor sleep patterns [35]. A systematic review by Coxon et al. [34] reported an association between pro-inflammatory diets, measured by the Dietary Inflammatory Index, with higher odds of sleep-related problems. However, these relationships may be bidirectional, as poor sleep may also reduce participation in physical activity and influence appetite, dietary choices, and meal timing [36,37].

The stress management domain was also associated with lower odds of sleep-related problems in the fully adjusted model, although the effect was modest. The stress management subscale of the U-SMILE captures behavioral responses to stress (i.e., relaxation practices and coping strategies) rather than perceived stress levels per se, which may attenuate the observed association. Additionally, stress and sleep share a well-established bidirectional relationship [38], meaning that the direction and magnitude of their association may be difficult to disentangle in a cross-sectional design. A two-wave longitudinal study of 302 undergraduate students supported this bidirectional relationship, with stress having a stronger impact on sleep problems (β = 0.345, *p* < 0.01) than sleep problems had on stress (β = 0.203, *p* < 0.01) [39]. Future longitudinal studies incorporating validated measures of both perceived stress and behavioral stress management practices would help clarify this relationship. From a health-promotion perspective, substance-use avoidance, social relationships, nutrition, and other non-sleep lifestyle domains may represent relevant areas for intervention among first-year university students, beyond sleep-hygiene behaviors already captured by the sleep quality domain. Strategies targeting these domains should be evaluated in future longitudinal and intervention studies.

### 4.2. Limitations and Strengths

Several limitations should be acknowledged when interpreting these results. First, the cross-sectional design precludes causal inference and does not exclude reverse causation or residual confounding. Longitudinal and intervention-based research is warranted to clarify the direction of these associations. Second, all study variables were self-reported, which may introduce recall and social desirability bias, particularly for domains such as substance use and sleep quality. Although the instruments used refer to recent, well-defined time periods, and were administered anonymously to minimize this risk, future studies would benefit from incorporating objective assessments, such as actigraphy to evaluate sleep patterns and accelerometry to measure physical activity. Third, the primary outcome was based on the DSM-5 Level 1 single-item sleep domain. Absolute proportion estimates and associations may therefore differ from studies that administered the PSQI to all participants. Fourth, the U-SMILE sleep quality domain and the Level 1 outcome are related constructs (habitual sleep behaviors versus a two-week symptom screen). Because of this overlap, the sleep quality domain was excluded from the primary domain-specific models; post-hoc sensitivity analyses showed that the global U-SMILE association remained after that domain was subtracted from the total score (Table S1); these analyses were not pre-specified. Fifth, the study was conducted at a single private university and included only first-year students from a predominantly Mestizo population (98.8% Mestizo), which may limit extrapolation to other university settings and student populations. Sixth, although all 3602 eligible first-year students were invited to participate, only 2007 completed the questionnaire, corresponding to a response rate of 55.7%. Because individual-level information was unavailable for nonrespondents, we could not compare their characteristics with those of participants. Therefore, non-response and selection bias cannot be excluded, as students who participated may have differed in their lifestyle behaviors, health characteristics, or sleep-related problems. Seventh, the stress management domain of the U-SMILE may not fully capture the complexity of the stress–sleep relationship because the subscale measures behavioral stress management practices rather than perceived stress levels. Future studies should complement the U-SMILE with validated perceived stress instruments to disentangle behavioral and psychological components of this pathway.

Despite these limitations, this study has several notable strengths. The large sample size, obtained through a census-based approach, enhanced statistical power and improved the precision of effect estimates. Additionally, the U-SMILE instrument used is a particular strength, as it operationalizes LM as a multidimensional construct encompassing seven validated lifestyle pillars rather than examining isolated behavioral risk factors, thereby aligning with the holistic framework of LM as defined by the ACLM. The DSM-5 Level 1 Cross-Cutting Symptom Measure has been evaluated in university populations, and defining the outcome at Level 1 maximized analytic completeness. The regression analyses were adjusted for multiple sociodemographic variables and health-related covariates, to account for potential confounding. Furthermore, the combined analytical strategy (multivariable logistic regression with predicted probability curves and formal testing for nonlinearity) allows for both adjusted effect estimation and the visualization of the LM–sleep gradient, thereby improving the robustness and interpretive value of the findings. Finally, this study addresses a meaningful gap in the literature, representing one of the first investigations to examine LM holistically in relation to sleep outcomes among university students in Ecuador and Latin America.

## 5. Conclusions

In this cross-sectional study of Ecuadorian university students, higher adherence to LM, as measured by the U-SMILE, was associated with lower odds of sleep-related problems defined by the DSM-5 Level 1 sleep domain. Among the individual domains, sleep, avoidance of substance use, and social relationships showed the strongest associations, while nutrition, nature engagement, physical activity, and stress management showed more modest associations. These findings highlight the potential value of a multidimensional LM approach for understanding sleep-related problems in university students. Future longitudinal and intervention studies are needed to further clarify these relationships and evaluate their implications for university health promotion.

## Figures and Tables

**Table 1:** Sample characteristics among Ecuadorian first-year university students.

Variable	Total (*N* = 2007)	Missing, *n* (%)
Age (years), median (IQR)	18.0 (18.0–19.0)	0 (0.0)
Age group (%)		
≤18 years old	1356 (67.6)	
>18 years old	651 (32.4)	
Sex (%)		0 (0.0)
Male	984 (49.0)	
Female	1023 (51.0)	
Sexual orientation (%)		0 (0.0)
Heterosexual	1748 (87.1)	
Non-heterosexual	259 (12.9)	
Race/ethnicity (%)		0 (0.0)
Non-Mestizo	24 (1.2)	
Mestizo	1983 (98.8)	
Marital status (%)		0 (0.0)
Single	1780 (88.7)	
Non-single	227 (11.3)	
Student accommodation (%)		0 (0.0)
Yes	70 (3.5)	
No	1937 (96.5)	
Work status (%)		1 (0.0)
Yes	292 (14.6)	
No	1714 (85.4)	
Field of study (%)		0 (0.0)
Health sciences	569 (28.4)	
Non-health sciences	1438 (71.6)	
BMI (kg/m^2^) (IQR)	22.5 (20.3–24.6)	16 (0.8)
BMI status (%)		
Underweight	177 (8.9)	
Normal weight	1395 (70.1)	
Overweight	358 (18.0)	
Obesity	61 (3.1)	
Mental disorder status (%)		0 (0.0)
No	1660 (82.7)	
Yes	347 (17.3)	
Physical condition status (%)		0 (0.0)
No	1373 (68.4)	
Yes	634 (31.6)	
Overall U-SMILE (score) (IQR)	65.0 (60.0–70.0)	7 (0.3)
Sleep-related problems (DSM-5 Level 1 sleep domain ≥ 2) (%)		101 (5.0)
No	994 (52.2)	
Yes	912 (47.8)	

BMI, body mass index; IQR, interquartile range; DSM-5, Diagnostic and Statistical Manual of Mental Disorders, Fifth Edition.

**Table 2:** Association between U-SMILE scores and sleep-related problems (DSM-5 Level 1 sleep domain ≥ 2) among Ecuadorian first-year university students.

Predictor	Model	*N*	OR per 1 Point (95% CI)	OR per 1 SD (95% CI)	*p*-Value
Overall U-SMILE	Model 0	1906	0.92 (0.91–0.94)	0.54 (0.49–0.60)	<0.001
Overall U-SMILE	Model 1	1906	0.92 (0.91–0.94)	0.55 (0.50–0.61)	<0.001
Overall U-SMILE	Model 2	1893	0.93 (0.91–0.94)	0.56 (0.50–0.62)	<0.001
Nutrition	Model 0	1906	0.91 (0.86–0.97)	0.87 (0.79–0.95)	0.002
Nutrition	Model 1	1906	0.90 (0.85–0.96)	0.85 (0.78–0.93)	<0.001
Nutrition	Model 2	1893	0.90 (0.85–0.96)	0.85 (0.78–0.94)	0.001
Avoidance of substance use	Model 0	1906	0.86 (0.81–0.90)	0.75 (0.68–0.82)	<0.001
Avoidance of substance use	Model 1	1906	0.85 (0.80–0.89)	0.73 (0.67–0.81)	<0.001
Avoidance of substance use	Model 2	1893	0.87 (0.83–0.92)	0.78 (0.70–0.86)	<0.001
Physical activity	Model 0	1906	0.91 (0.88–0.95)	0.80 (0.73–0.88)	<0.001
Physical activity	Model 1	1906	0.92 (0.89–0.96)	0.82 (0.75–0.91)	<0.001
Physical activity	Model 2	1893	0.93 (0.89–0.97)	0.85 (0.76–0.94)	0.002
Stress management	Model 0	1906	0.97 (0.91–1.02)	0.95 (0.87–1.04)	0.264
Stress management	Model 1	1906	0.97 (0.91–1.02)	0.95 (0.87–1.04)	0.237
Stress management	Model 2	1893	0.93 (0.88–0.99)	0.89 (0.81–0.98)	0.021
Social relationships	Model 0	1906	0.89 (0.87–0.92)	0.70 (0.64–0.77)	<0.001
Social relationships	Model 1	1906	0.89 (0.86–0.92)	0.69 (0.63–0.76)	<0.001
Social relationships	Model 2	1893	0.90 (0.87–0.93)	0.71 (0.64–0.78)	<0.001
Engagement with the natural environment	Model 0	1906	0.91 (0.87–0.95)	0.82 (0.75–0.90)	<0.001
Engagement with the natural environment	Model 1	1906	0.90 (0.86–0.94)	0.80 (0.73–0.88)	<0.001
Engagement with the natural environment	Model 2	1893	0.91 (0.87–0.95)	0.82 (0.75–0.91)	<0.001

OR, odds ratio; CI, confidence interval; SD, standard deviation; DSM-5, Diagnostic and Statistical Manual of Mental Disorders, Fifth Edition. Model 0 = crude. Model 1 = adjusted for age and sex. Model 2 = fully adjusted (age, sex, sexual orientation, race/ethnicity, marital status, student accommodation, work status, degree program, body mass index status, mental disorder status, physical condition status). Multivariable logistic regression models. OR per 1 SD uses the sample SD of the predictor in the corresponding complete-case model sample.

## Data Availability

The data presented in this study are available on request from the corresponding author. The data are not publicly available due to privacy and ethical restrictions related to research on human subjects.

## Supplementary Materials

The following supporting information can be downloaded at https://www.mdpi.com/article/10.3390/healthcare14182891/s1, Table S1: Post-hoc sensitivity analysis: Overall U-SMILE score with and without the sleep quality domain Figure S1: Assessment of linearity between U-SMILE scores (overall and domains) and sleep-related problems.

## Abbreviations

The following abbreviations are used in this manuscript:

AASM	American Academy of Sleep Medicine
ACHA-NCHA	American College Health Association-National College Health Assessment
ACLM	American College of Lifestyle Medicine
AIC	Akaike information criterion
BIC	Bayesian information criterion
BMI	Body mass index
CEISH-UDLA	Comité de Ética para la Investigación con Seres Humanos de la Universidad de las Américas
CI	Confidence interval
DSM-5	Diagnostic and Statistical Manual of Mental Disorders, Fifth Edition
IQR	Interquartile range
LM	Lifestyle Medicine
OR	Odds ratio
PSQI	Pittsburgh Sleep Quality Index
Q-Q	Quantile-quantile
REDCap	Research Electronic Data Capture
REM	Rapid eye movement
SMILE	Short Multidimensional Inventory on Lifestyle Evaluation
STROBE	Strengthening the Reporting of Observational Studies in Epidemiology
UDLA	Universidad de Las Américas
UNILIFE-M	UNIversity students’ LIFEstyle behaviours and Mental health cohort
U-SMILE	Short Multidimensional Inventory Lifestyle Evaluation for University Students
