# Supplementary material for: Adherence to Lifestyle Medicine and Sleep-Related Problems Among First-Year University Students in Quito, Ecuador, 2024–2025: A Cross-Sectional Study
Source: Healthcare (Basel). 2026 Sep 8;14(18):2891. doi: 10.3390/healthcare14182891 (PMC13606756; doi:10.3390/healthcare14182891)
Supplement: Supplementary file 1 [file healthcare-14-02891-s001.zip › healthcare-4461621-supplementary.pdf]

Supplementary material

Table S1. Post-hoc sensitivity analysis: U-SMILE global score with and without the sleep quality domain.

Predictor	Model	N	OR per 1 point (95% CI)	OR per 1 SD (95% CI)	p-Value
Overall U-SMILE	Model 0	1906	0.92 (0.91–0.94)	0.54 (0.49–0.60)	<0.001
Overall U-SMILE	Model 1	1906	0.92 (0.91–0.94)	0.55 (0.50–0.61)	<0.001
Overall U-SMILE	Model 2	1893	0.93 (0.91–0.94)	0.56 (0.50–0.62)	<0.001
Overall U-SMILE (excluding sleep domain)	Model 0	1906	0.94 (0.92–0.95)	0.64 (0.58–0.71)	<0.001
Overall U-SMILE (excluding sleep domain)	Model 1	1906	0.94 (0.92–0.95)	0.64 (0.59–0.71)	<0.001
Overall U-SMILE (excluding sleep domain)	Model 2	1893	0.94 (0.93–0.95)	0.66 (0.60–0.73)	<0.001

OR, odds ratio; CI, confidence interval; SD, standard deviation. Model 0 = crude. Model 1 = adjusted for age and sex. Model 2 = fully adjusted as in the main analyses. These analyses were not pre-specified. OR per 1 SD uses the sample SD of the predictor in the corresponding complete-case model sample. The excluded sleep domain comprises U-SMILE items on habitual sleep duration, regularity, and restorative sleep.

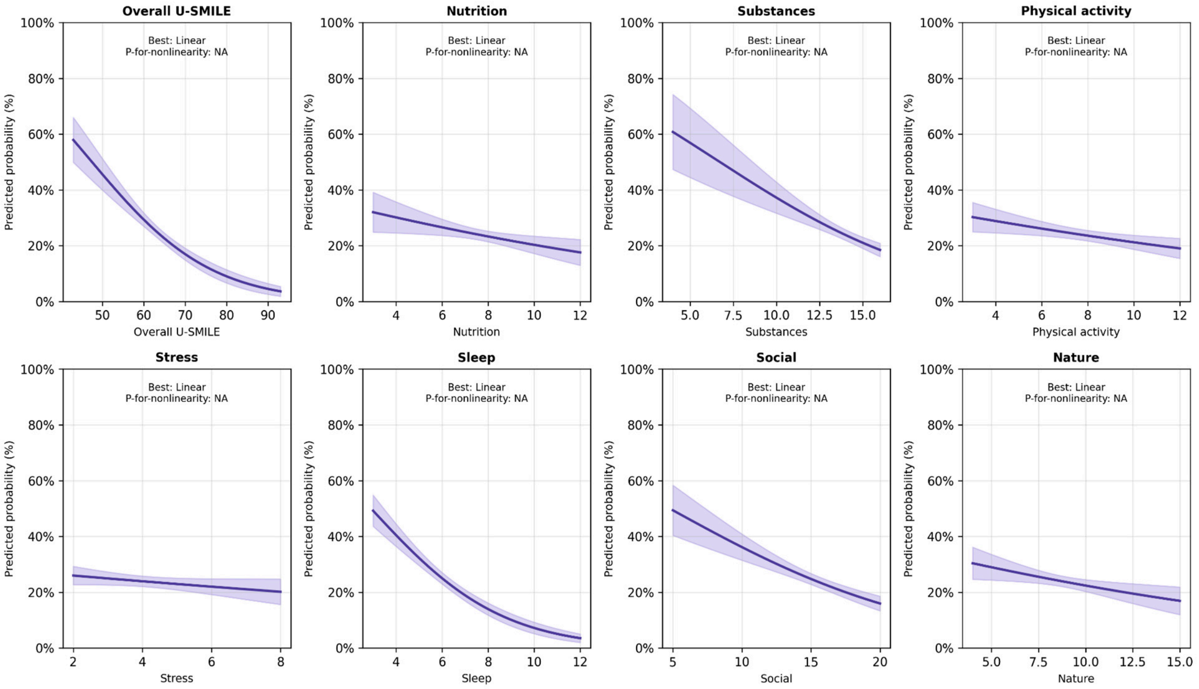

Figure S1. Assessment of linearity between U-SMILE scores (overall and domains) and sleep-related problems (DSM-5 Level 1 sleep domain ≥2).
